# Systolic pulmonary artery pressure threshold to define pulmonary and peripheral congestion in acute heart failure in absence of severe tricuspid regurgitation

**DOI:** 10.3389/fcvm.2025.1678712

**Published:** 2025-09-23

**Authors:** Maria Giulia Bellicini

**Affiliations:** Institute of Cardiology, ASST Spedali Civili of Brescia, Brescia, Italy

**Keywords:** acute heart failure, echocardiographic cut off, systolic pulmonary artery pressure threshold, peripheral congestion, lung congestion

## Abstract

**Background and aims:**

Pulmonary and/or peripheral venous congestion defines the clinical diagnosis of acute heart failure (AHF). However, the systolic pulmonary arterial pressure (sPAP) thresholds at which pulmonary (chest x-ray) and inferior vena cava (IVC) congestion occur are not well established. This study aimed to identify a cut-off value of sPAP that reliably indicates AHF.

**Methods and results:**

We retrospectively included 380 consecutive patients hospitalized for AHF at an Italian referral centre, after excluding those with severe tricuspid regurgitation. Receiver operating characteristic (ROC) curve analysis and Youden's J statistic identified a threshold of sPAP ≥ 48.75 mmHg as the most accurate in predicting both pulmonary (sensitivity = 89.9%, specificity = 73%) and peripheral (sensitivity = 88.3%, specificity = 82.5%) fluid overload. The association between this sPAP threshold and both pulmonary and peripheral congestion was confirmed by chi-square testing (*p* < 0.001) and multivariate logistic regression (*p* < 0.001). After adjustment for confounders, sPAP ≥ 49 mmHg was independently associated with all-cause death or heart failure (HF) hospitalization (HR = 1.713; 95% CI 1.127–2.602; *p* = 0.012).

**Conclusions:**

sPAP threshold of 49 mmHg identifies congestion with clinically useful accuracy—pulmonary (chest X-ray) congestion.

## Introduction

Heart failure (HF) is a complex clinical syndrome resulting from structural and/or functional cardiac abnormalities that impair the ability of the ventricle to fill with or eject blood, leading to symptoms such as dyspnoea and fatigue, and signs of fluid retention (1). In most cases, dysfunction involves the left heart and leads to progressive fluid accumulation, resulting in pulmonary and systemic venous congestion. On echocardiographic evaluation, affected patients often show inferior vena cava (IVC) congestion and elevated systolic pulmonary artery pressure (sPAP).

According to invasive and echocardiographic data from healthy populations, the upper limit of normal for sPAP is approximately 30 mmHg (2, 3). However, in HF cohorts thresholds have been inconsistent. Some studies reported prognostic associations of sPAP values above 35–40 mmHg with increased risk of rehospitalization and mortality (4–6), while others proposed stratified categories such as mild (35–49 mmHg), moderate (50–59 mmHg), and severe (≥60 mmHg) pulmonary hypertension (7). Importantly, the 2019 ESC consensus on the use of diuretics in HF did not define any sPAP-based threshold for congestion, instead emphasizing clinical signs, natriuretic peptides, and imaging findings including IVC dilatation and chest x-ray abnormalities (8). As a result, no diagnostic cut-off has been validated to distinguish compensated from decompensated HF, particularly in acute settings.

The presence of predominant right-sided congestion in some patients has likely further complicated the interpretation of sPAP values, limiting the identification of a universally accepted threshold (9, 10).

This study was designed to test the hypothesis that a clinically meaningful sPAP cut-off can accurately identify pulmonary and peripheral congestion and predict adverse outcomes in patients with AHF.

## Methods

### Study design and patient selection

A total of 819 consecutive patients admitted for acute heart failure (HF) to the Cardiology Department of a major Italian referral center (Spedali Civili of Brescia) between January 2022 and November 2023 were retrospectively identified. The diagnosis of AHF was established according to the European Society of Cardiology (ESC) criteria.

**Figure 1:**
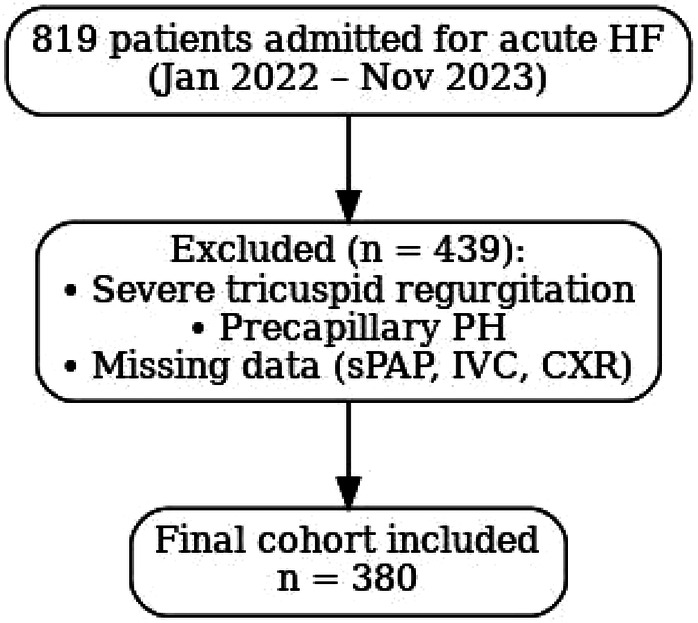
Flow diagram of patient selection. Flow diagram of patient selection. A total of 819 patients with acute HF were screened; 380 patients were included after exclusions (severe TR, precapillary PH, or missing data).

Major exclusion criteria were:

1. severe tricuspid regurgitation, because in this setting patients may develop isolated right-sided fluid accumulation, with peripheral congestion without pulmonary congestion, and therefore may show normal pulmonary artery systolic pressures despite being clinically fluid-overloaded. In addition, in massive or torrential tricuspid regurgitation, right atrial pressure cannot be reliably estimated from IVC size and collapsibility, as the IVC is invariably dilated and non-collapsible due to regurgitant flow, even when actual central venous pressures are low (9, 10);
2. precapillary pulmonary hypertension; and
3. missing data for key variables of interest (sPAP, IVC, or chest x-ray). After applying these criteria, 380 patients were included in the final analysis (Figure 1).

For each patient, demographic and baseline clinical characteristics, comorbidities, vital signs, physical exam findings, cardiac rhythm, laboratory results, chest x-ray, and echocardiographic parameters at admission were reviewed. Prognostic outcomes—including HF rehospitalization and all-cause mortality—were also recorded. All data were collected anonymously.

### Echocardiography

A comprehensive transthoracic echocardiogram at rest was performed upon presentation to the emergency department or shortly after admission to the cardiology ward. Studies were conducted by experienced operators using Philips Affiniti or Epiq ultrasound systems with a 1–5 MHz matrix array sector probe.

Left and right ventricular dimensions and function, as well as the severity of valvular regurgitation, were assessed using standard two- and four-chamber views, in accordance with ESC recommendations (11). Left ventricular diastolic function was evaluated by pulsed Doppler analysis of transmitral inflow, specifically the E/A wave ratio.

Systolic pulmonary artery pressure (sPAP) was estimated according to the ESC/ERS Pulmonary Hypertension Guidelines, from the peak velocity of the tricuspid regurgitation (TR) jet using the simplified Bernoulli equation [sPAP = 4 × (TRV)^2^ + right atrial pressure]. Right atrial pressure (RAP) was derived from inferior vena cava (IVC) diameter and respiratory collapsibility, expressed in increments of 5 mmHg, using guideline-recommended thresholds (12). This non-invasive echocardiographic method is the current standard approach to estimate pulmonary pressures.

All patients also underwent a core-laboratory echocardiogram within 72 hours of admission, pulmonary pressure estimates from both studies were reviewed, and the higher value was retained to reduce the risk of underestimation.

### Chest radiograph

A chest x-ray was performed at admission in all patients. Pulmonary congestion was defined as the presence of pleural effusion, accentuation of the interstitial–vascular markings, or congestion of at least one pulmonary hilum (unilateral or bilateral).

In patients with known chronic obstructive or restrictive pulmonary disease, or chronic pleural disease, the chest x-ray was considered diagnostic of AHF decompensation only if acute pleural effusion was present or if acute interstitial changes were observed. In all other cases, findings were deemed non-specific (13).

### Statistical analysis

Baseline characteristics were expressed as frequencies and percentages for categorical variables, and as means with standard deviations for continuous variables. Comparisons between groups were performed using chi-square or Levene's test, as appropriate.

Patient characteristics, comorbidities, and cardiovascular outcomes were extracted from the hospital database and regional electronic health records. Univariate binary logistic regression was used to assess associations between baseline variables and the presence of pulmonary congestion on chest x-ray.

Variables found to be statistically significant in univariate analysis were then evaluated using receiver operating characteristic (ROC) curves and Youden's J statistic to identify optimal thresholds predictive of congestion. The resulting sPAP threshold was subsequently tested for its ability to predict IVC (peripheral) congestion using ROC analysis.

Multivariate logistic regression was performed to identify independent predictors of chest x-ray congestion. Clinically relevant variables and those significant in univariate analysis (*p* < 0.05) were included, unless they exhibited multicollinearity with sPAP or CVP. Specifically, NYHA class, peripheral edema, lung rales and mitral regurgitation grade were excluded from the final model to preserve statistical validity. Collinearity was assessed through inspection of standard errors and confidence intervals; no relevant multicollinearity was detected.

The prognostic significance of the identified sPAP cut-off was assessed using Cox proportional hazards models for the outcomes of all-cause mortality and the composite of cardiovascular and heart failure hospitalizations. All analyses were conducted using SPSS statistical software.

## Results

### Patients’ baseline characteristics

Baseline characteristics of the study population are summarized in Table 1 and Supplemetary Table S1. The mean age was 76.3 years (± 11.2), and 34.7% of patients were female. The mean left ventricular ejection fraction (LVEF) was 38.2% (± 13.4), with a mean systolic pulmonary artery pressure (sPAP) of 51.6 mmHg (± 13 mmHg) and a central venous pressure (CVP) of 10.8 mmHg (± 4.5 mmHg). Pulmonary congestion on chest x-ray was present in 72.1% of patients. The mean NT-proBNP level was 8,258 pg/ml (± 9,271 pg/ml).

**Table 1:** Baseline characteristics of the study population.

Variable	Value ± SD/frequency (%)
	*N* = 380
Demographics and comorbidities	
Age at inclusion (years)	76,3 ± 11,2
Female sex	132 (34,7%)
Hypertension	259 (68,1%)
Dyslipidemia	201 (52,9%)
Diabetes mellitus	125 (32,9%)
Permanent AF	88 (23,1%)
Prior CAD diagnosis	122 (32,1%)
Prior valve surgery	39 (10,2%)
Prior percutaneous valve intervention	22 (5,7%)
Objective examination	
NYHA class III	143 (37,6%)
NYHA class IV	146 (38,4%)
Declive oedema	163 (42,9%)
Lung rales	169 (44,4%)
Vital parameters	
SBP (mmHg)	138,8 ± 32,7
HR(bpm)	88,64 ± 25,856
Rhythm at ECG	
Sinus rhythm	260 (68,4%)
AF	120 (31,6%)
Echocardiogram	
LVEF(%)	38,23 ± 13,396
LA volume(ml)	108,08 ± 64,295
RV disfunction	47 (12,4%)
Severe MR	160 (42,1%)
Severe MS	4 (1,0%)
Severe AS	34 (8,9%)
Severe AR	17 (4,4%)
sPAP (mmHg)	51,58 ± 12,98
CVP (mmHg)	10,81 ± 4,49
Lung congestion at chest x-ray	274 (72,1%)
Laboratory exams	
NTproBNP (pg/ml)	8258,35 ± 9271,41
Troponin T (ng/L)	532,90 ± 2689,84
Creatinine (mg/dl)	1,66 ± 1,14
Hemoglobin (g/dl)	12,13 ± 2,20
ALT (UI/L)	59,68 ± 215,41

Baseline demographic, clinical, and echocardiographic characteristics of the study population are reported. Continuous variables are expressed as mean ± standard deviation; categorical variables as percentages.

ALT, alanine aminotransferase; AF, atrial fibrillation; AR, aortic regurgitation; AS, aortic stenosis; CAD, coronary artery disease; CVP, central venous pressure; HR, heart rate; LA, left atrium; LVEF, left ventricular ejection fraction; MR, mitral regurgitation; MS, mitral stenosis; NTproBNP, N-terminal pro-B-type natriuretic peptide; NYHA, New York heart association; SBP, systolic blood pressure; SD, standard deviation; sPAP, systolic pulmonary artery pressure; TnT, troponin T.

### Characteristics of the study population stratified by chest-x-ray congestion

Patients with evidence of congestion on chest x-ray at admission were more likely to present with higher weight (OR = 0.978, 95% CI: 0.963–0.994, *p* = 0.006), NYHA class III or IV (OR = 2.674, 95% CI: 1.914–3.737, *p* < 0.001), declive oedema (OR = 6.375, 95% CI: 3.607–11.269, *p* < 0.001), severe mitral regurgitation (OR = 2.528, 95% CI: 1.546–4.134, *p* < 0.001), elevated sPAP (OR = 1.13, 95% CI: 1.098–1.163, *p* < 0.001), and increased CVP (OR = 1.406, 95% CI: 1.304–1.516, *p* < 0.001). Laboratory findings associated with congestion included NT-proBNP > 3,000 pg/ml (OR = 5.655, 95% CI: 3.212–9.958, *p* < 0.001), troponin T > 14 ng/L (OR = 4.136, 95% CI: 1.570–10.894, *p* = 0.004), and higher ALT levels (OR = 1.013, 95% CI: 1.002–1.024, *p* = 0.02) (Supplemetary Table S2). After adjustment in a multivariate binary logistic regression, echocardiographic parameters and troponin T remained significantly associated with chest x-ray congestion (Table 2). No significant differences were observed between groups in terms of other clinical characteristics, comorbidities, or preadmission therapy (Supplementary Table S2).

**Table 2:** Multivariate logistic regression for the presence of pulmonary congestion on chest x-Ray.

Variable	*P* value	HR (95% C.I.)
Female sex	0,737	0,821 (0,259; 2,598)
Age	0,129	0,956 (0,902;1,013)
Weight (kg)	0,004	0,955 (0,925; 0,985)
Hypertension	0,064	3,162 (0,936; 10,678)
LVEF (%)	0,659	1,010 (0,966; 1,057)
sPAP (mmHg)	0,006	1,083 (1,023; 1,145)
CVP (mmHg)	0,001	1,332 (1,121; 1,583)
Troponin T > 14 ng/L	0,027	9,162 (1,283; 65,447)
NTproBNP >3,000 mg/dl	0,102	2,420 (0,840; 6,971)
ALT (UI/L)	0,468	1,007 (0,989; 1,026)

Variables independently associated with chest x-ray pulmonary congestion are shown. Odds ratios [Exp(B)] are reported with 95% confidence intervals. Variables were selected based on clinical relevance and univariate significance; collinear variables were excluded.

### sPAP cut off

**Figure 2:**
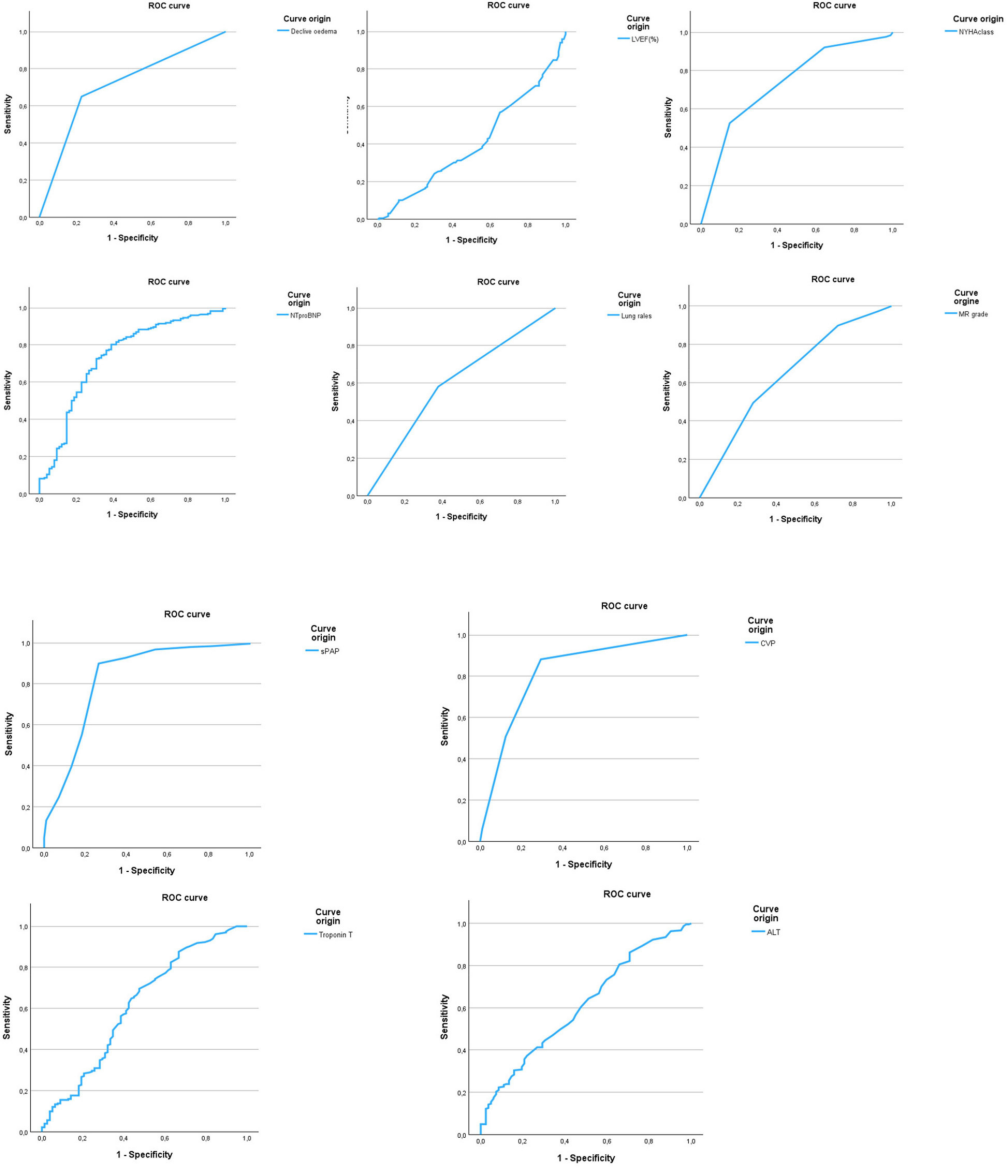
Receiver operating characteristic (ROC) curves of parameters predicting chest x-ray congestion. ROC curves are shown for all variables significantly associated with chest x-ray pulmonary congestion in univariate analysis. sPAP demonstrated the highest diagnostic performance (AUC = 0.823), while LVEF showed no discriminatory value (AUC = 0.407). Optimal cut-off values were identified using 9.

A receiver operating characteristic (ROC) curve analysis combined with Youden's J statistic was used to evaluate the diagnostic performance of variables significantly associated with chest x-ray pulmonary congestion. Among them, systolic pulmonary artery pressure (sPAP) demonstrated the best discriminatory ability. The analysis identified an optimal threshold of 48.75 mmHg. Adjacent values within the range 47.5–50 mmHg yielded nearly identical diagnostic performance (sensitivity 89.9%, specificity 73% with an ACU of 0.823); therefore, the cut-off is reported as ≈49 mmHg for clinical applicability (Table 3, Figure 2).

**Table 3:** Diagnostic performance of variables associated with chest x-ray congestion.

Variable	AUC	Sensitivity	Specificity	More performing cut off value	Youden test
NYHA class	0,736	0,885	0,621	3	0,508
Declive oedema (1/0)	0,713	0,65	0,232	1	0,118
Lung rales (1/0)	0,602	0,58	0,623	1	0,204
LVEF (%)	0,407				
MR grade	0,641	0,494	0,721	severe	0,215
sPAP (mmHg)	0,823	0,899	0,735	(47,5–50)	0,634
CVP (multiple of 5; mmHg)	0,816	0,881	0,708	10	0,589
Troponin T (ng/L)	0,614	0,695	0,526	34,5 ± 0,5	0,221
NTproBNP (pg/ml)	0,735	0,725	0,693	3,124 ± 25	0,419
ALT (UI/L)	0,61	0,372	0,98	32,5 ± 0,5	0,153

**Figure 3:**
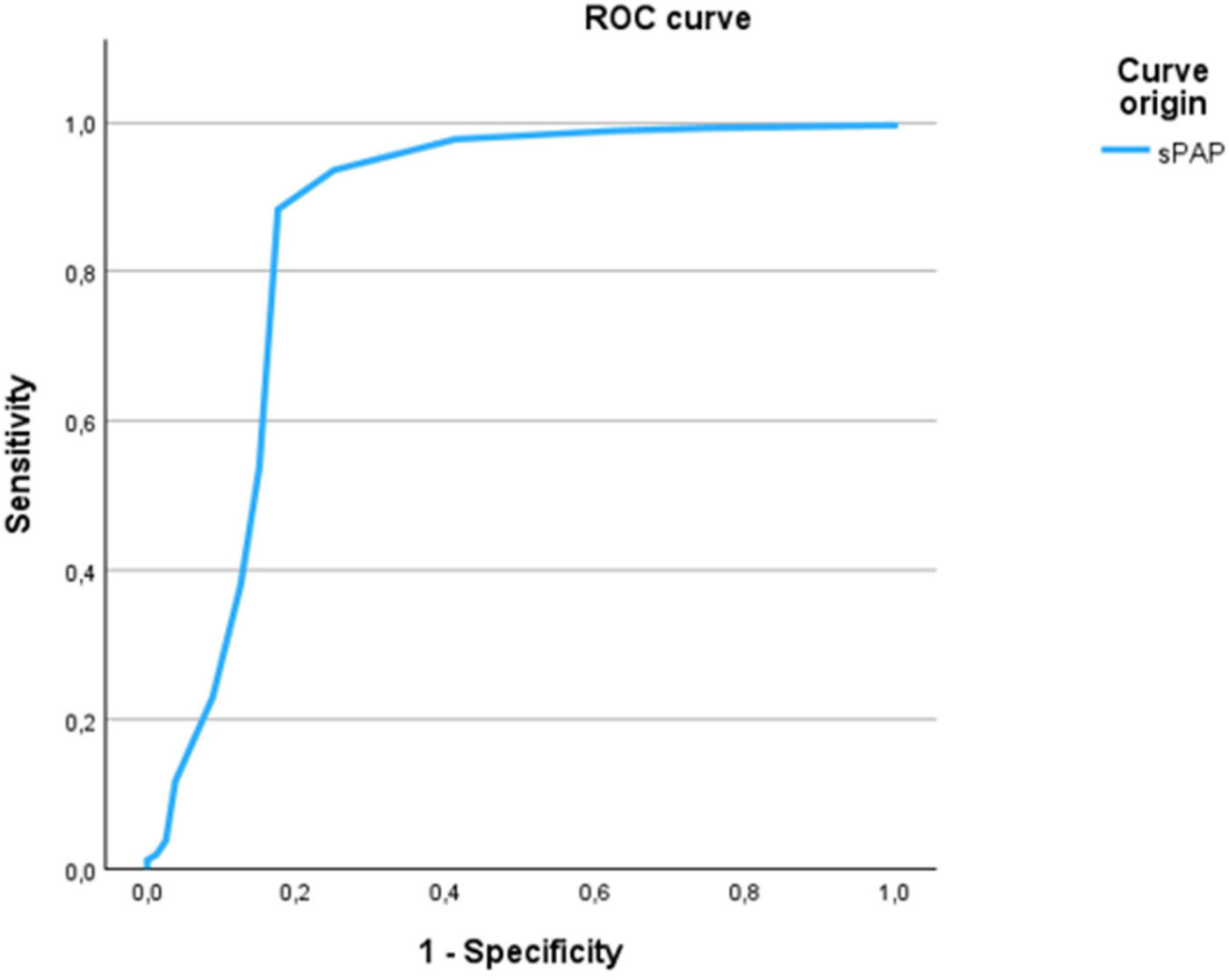
ROC curve of sPAP for the prediction of peripheral venous congestion. The ROC curve illustrates the discriminatory ability of sPAP to predict peripheral venous congestion as assessed by echocardiography. The optimal cut-off value of ≥48.75 mmHg yielded a sensitivity of 88.3% and a specificity of 82.5%, with an area under the curve (AUC) of 0.857.

The sPAP cut-off of 49 mmHg also showed strong performance in predicting peripheral venous congestion on echocardiography, with a sensitivity of 88.3% and specificity of 82.5% with an AUC of 0.857 (Figure 3). This threshold was further supported by chi-square testing and multivariate linear regression, which confirmed a significant association between sPAP ≥ 49 mmHg and both peripheral venous and chest x-ray pulmonary congestion (*p* < 0.001).

### Prognostic significance of sPAP cutoff

**Figure 4:**
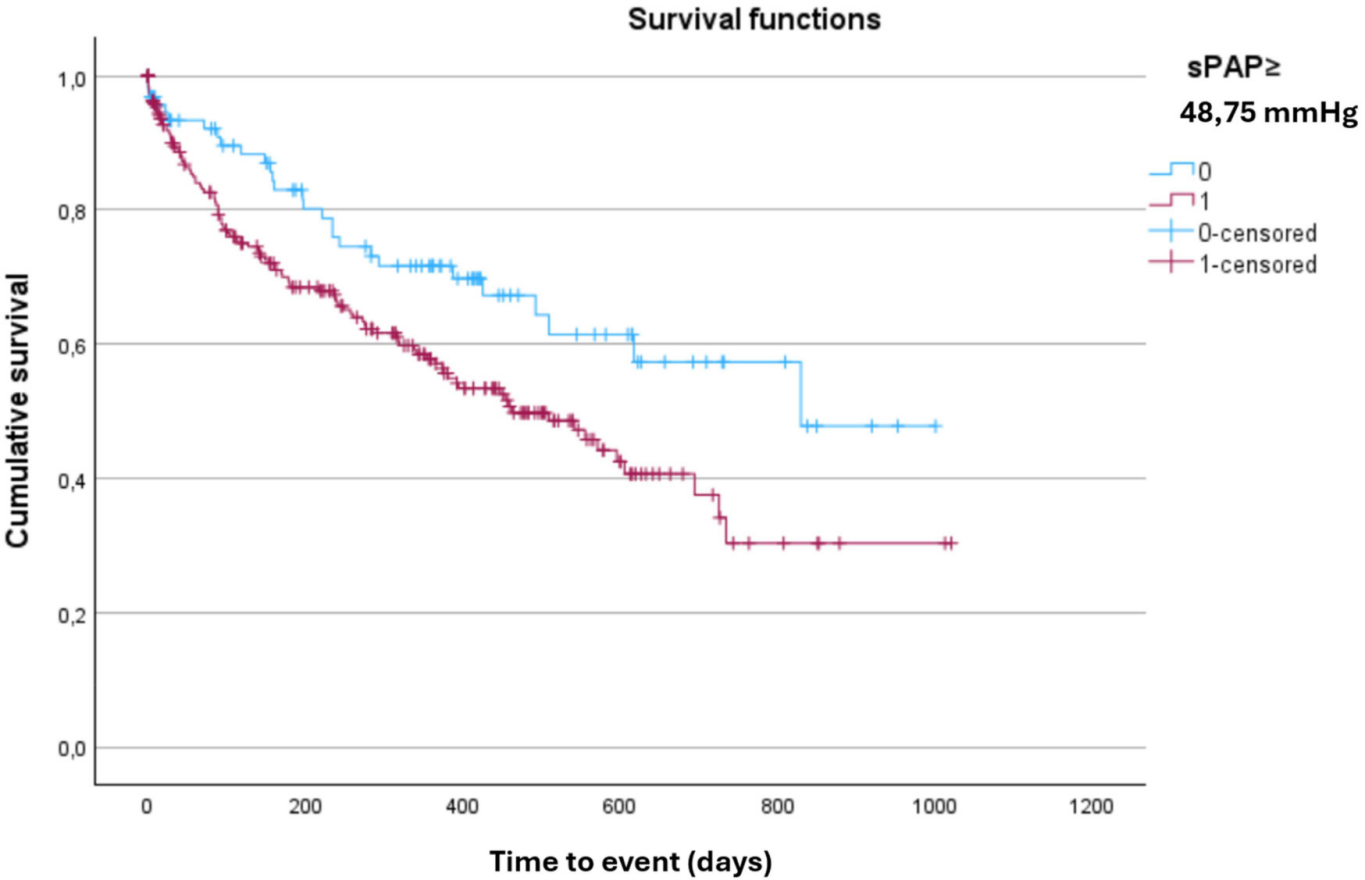
Kaplan–Meier curve for the composite outcome of all-cause death or heart failure hospitalization, stratified by sPAP Cut-Off. Kaplan–Meier analysis showing the probability of survival free from all-cause death or heart failure hospitalization according to sPAP ≥ 48.75. Patients above this threshold had significantly worse outcomes during follow-up (log-rank *p* < 0.05).

Overall, 25% of patients died and 15% required rehospitalization for HF. The combined endpoint of all-cause death or HF hospitalization occurred in 38% of patients over a median follow-up of 521 days (95% CI: 455–586 days). In univariable analysis, sPAP value ≥ 49 mmHg at admission was significantly associated with an increased risk of the composite outcome (*p* = 0.019) and with first cardiovascular hospitalization (*p* = 0.030). Survival analysis using the Kaplan–Meier method demonstrated a significantly worse prognosis in patients with sPAP ≥ 49 mmHg (Figure 4).

**Figure 5:**
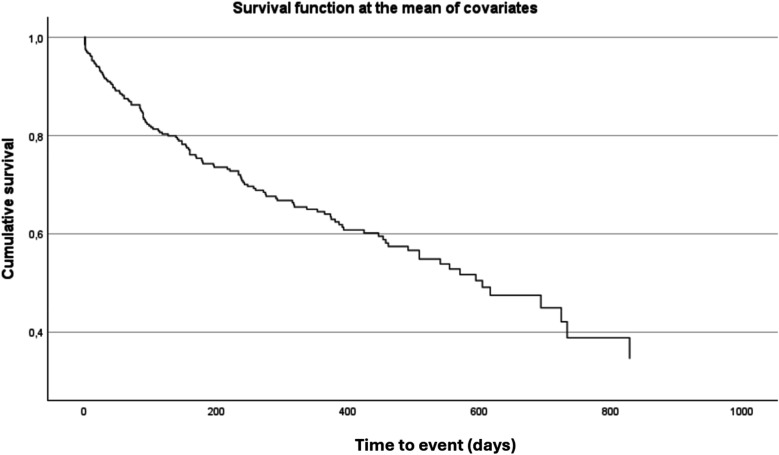
Forest plot from cox regression for the composite outcome of all-cause death or heart failure hospitalization. Forest plot displaying hazard ratios (HRs) with 95% confidence intervals for variables included in the multivariable Cox regression analysis. sPAP ≥ 48.75 was independently associated with an increased risk of the composite outcome (HR = 1.713, 95% CI: 1.127–2.602; *p* = 0.012). Age and sex also showed significant associations. LVEF was not independently predictive of outcome.

After multivariable adjustment, sPAP ≥ 49 mmHg at admission remained independently associated with an increased risk of the composite outcome of all-cause death or HF hospitalization (HR = 1.713; 95% CI: 1.127–2.602; *p* = 0.012) (Table 4, Figure 5).

**Table 4:** Cox regression for composite outcome all-cause death or HF hospitalization.

Variable	*P* value	HR (95% C.I.)
sPAP ≥ 48,75 mmHg	0,021	1,672 (1,082; 2,584)
Female sex	0,041	0,660 (0,442; 0,984)
Age (years)	0,002	1,029 (1,011; 1,049)
LVEF (%)	0,420	0,994 (0,980; 1,009)

## Discussion

Current Pulmonary Arterial Hypertension guidelines define 30 mmHg as the upper limit of normal for sPAP in healthy individuals (12). However, the 2019 ESC consensus on the use of diuretics in heart failure don't establish specific sPAP thresholds for defining euvolemia or congestion. Instead, fluid status is primarily evaluated based on clinical signs such as peripheral edema, natriuretic peptide levels (e.g., NT-proBNP), and imaging findings including IVC congestion and chest x-ray abnormalities (8).

To date, no study has clearly defined sPAP ranges for decompensated HF. The literature also shows disagreement regarding the definition of normal vs. pathological sPAP values, particularly in patients with comorbidities. For instance, Fisher and colleagues proposed stratified thresholds for pulmonary hypertension: mild (35–49 mmHg), moderate (50–59 mmHg), and severe (≥60 mmHg) (5–7). Similarly, McQuillan et al. reported that only 28% of 3,790 echocardiographically normal individuals had an sPAP <30 mmHg. They also noted that the upper reference limit might reach 40 mmHg in older adults or patients with obesity or heart failure (4).

Establishing sPAP cut-off values is challenging due to multiple confounders. One major issue is the frequent presence of severe tricuspid regurgitation in patients with decompensated HF, which often leads to IVC regardless of the patient's true volume status. Additionally, isolated right-sided congestion patterns do not always present with radiographic pulmonary congestion, further complicating the assessment.

Another limitation in the literature stems from high inter-operator and inter-centre variability in echocardiographic evaluation, particularly in emergency settings. Many studies lack complete imaging data at the time of admission, reducing diagnostic consistency. In contrast, our study was conducted in a high-volume tertiary care center with comprehensive clinical and echocardiographic assessments performed at admission and during hospitalization.

By applying stringent exclusion criteria, we found that a moderately elevated sPAP value—specifically, ≥49 mmHg—was highly sensitive and specific for identifying both pulmonary radiographic and peripheral venous congestion in patients with AHF. Our findings suggest that the proposed cut-off has potential clinical relevance, which may be confirmed by future validation in larger and prospective cohorts.In clinical practice, several alternative tools are available to assess congestion because clinical signs are not sensitive. Biomarkers such as NT-proBNP remain guideline-endorsed as sensitive rule-out tests for HF (13). However, their high inter-patient variability in the acute setting hampers the identification of reliable cut-offs with both high sensitivity and specificity, as also reflected in our ROC curve analysis. Echocardiographic indices such as E/e′ provide indirect estimates of filling pressures, but they are strongly influenced by technical and patient-related factors and often show limited reproducibility in emergency settings (14).

Lung ultrasound (LUS) has emerged as a highly sensitive technique, with B-lines closely correlating with pulmonary capillary wedge pressure Nevertheless, its specificity is limited, as B-lines may also occur in non-cardiac pulmonary diseases such as fibrosis or pneumonia (15, 16). In our retrospective cohort, LUS data were not systematically available. For this reason, we used chest x-ray as the reference standard. To increase its specificity, patients with chronic lung disease or pleural abnormalities were excluded unless acute interstitial changes or effusion were present. At the same time, sensitivity was preserved by including early radiographic signs such as hilar vascular congestion, which are known to appear at wedge pressures as low as ∼15 mmHg (8, 17). With these precautions, the diagnostic yield of chest x-ray may approximate the sensitivity of B-lines while maintaining greater specificity under stringent criteria.

## Limitations

This study has limitations. Its retrospective, single-center design may limit generalizability. Echocardiographic estimation of pulmonary pressures, although performed by experienced operators using high-quality equipment and guideline-based methods, is inherently subject to variability. All patients also underwent a core-laboratory echocardiogram within 72 hours, findings from both studies were considered, and the higher PASP value was retained. This approach was intended to minimize misclassification and to strengthen confidence in the echocardiographic assessment of pulmonary congestion. Invasive hemodynamic validation (e.g., right heart catheterization with pulmonary capillary wedge pressure measurement) was not available, preventing direct comparison of echocardiographic and invasive pressure estimates. sPAP was discretized in 5 mmHg steps, which yields stepwise ROC curves; results were robust across cut-offs within 47.5–50.0 mmHg. Although chest x-ray is widely available and routinely used in the acute setting, it is not the gold standard for assessing pulmonary congestion; its sensitivity is lower than lung ultrasound or invasive pressure monitoring, and it may be affected by concomitant lung disease. To mitigate this limitation, we applied strict diagnostic criteria and excluded patients with chronic pulmonary conditions that could confound interpretation. Finally, although exclusion criteria (e.g., severe tricuspid regurgitation, precapillary pulmonary hypertension, missing key data) were clinically and methodologically justified, they may have introduced some degree of selection bias, limiting the representativeness of the study cohort.

## Conclusion

In this study, we identified a systolic pulmonary arterial pressure (sPAP) threshold of ≥49 mmHg as a reliable marker of AHF. This value was independently associated with both objective signs of congestion and adverse clinical outcomes, supporting its potential clinical utility in routine assessment. Prospective multicentre studies are warranted to validate this threshold and to further define its role in guiding clinical management.

## Data Availability

The raw data supporting the conclusions of this article will be made available by the authors, without undue reservation.
