# Supplementary material for: Systolic pulmonary artery pressure threshold to define pulmonary and peripheral congestion in acute heart failure in absence of severe tricuspid regurgitation
Source: Front Cardiovasc Med. 2025 Sep 23;12:1678712. doi: 10.3389/fcvm.2025.1678712 (PMC12500580; doi:10.3389/fcvm.2025.1678712)
Supplement: Supplementary file 1 [file Datasheet1.pdf]

Supplementary Table S1. Full Clinical, Echocardiographic, and Laboratory Data
This table includes additional baseline variables not shown in Table 1.

Variable	Value (SD) / Frequency (%)
**Demographics and comorbidities**	
Age at inclusion (years) ±standard deviation (SD)	76,3 ± 11,2
Height (cm)	167,65 ± 9,253
Weight(kg)	74,55 ± 17,33
Time since HF diagnosis (months)	30,14 ± 62,14
Number of HF hospitalizations in last year	0,71 ± 6,130
Time since last HF hospitalization (months)	12,65 ± 27,41
Episodes of congestion requiring IV diuretics in last year	0,18 ± 0,38
Episodes of low output requiring inotropes/vasopressors in last year	0,03 ± 0,16
Smoker active/former	91 (23,9%)
Peripheral artery disease	70 (18,4%)
Prior stroke/TIA	53 (13,9%)
Prior AF	152 (40%)
Prior MI	104 (27,4%)
Prior PCI	99 (26,0%)
Prior CABG	26 (6,84%)
Prior valve surgery	
○ MV repair	5 (1,3%)
○ MV replacement	10 (2,6%)
○ AV repair	1 (0,2%)
○ AV replacement	21 (5,5%)
○ TV repair	0
○ other	1 (0,2%)
Prior percutaneous valve intervention	
○ MitraClip	5 (1,3%)
○ TAVR	15 (3,9%)
Prior myocarditis	2 (0,5%)
Known cardiomiopathy	
○ Dilated CM	37 (9,7%)
○ Hypertrophic CM	3 (0,8%)
Prior device implantation	
○ PM	40 (10,5%)
○ ICD	27 (7,1%)
○ CRTD	19 (5%)
○ CRTP	3 (0,8%)
COPD	43 (11,3%)
CKD	162 (42,6%)
Dialysis	6 (1,6%)
History of cancer	70 (18,4%)
Depressive disorder	25 (6,6%)
Dementia	49 (12,9%)
De novo HF	181 (47,6%)
**Objective examination**	
○ NYHA class I	4 (1,0%)
○ NYHA class II	42 (11,0%)
**Vital parameters**	
DBP (mmHg)	78,16 ± 20,0

MAP (mmHg)	84,7 ± 40,1
**Echocardiogram**	
EDV(mL)	144,37 ± 65,530
EDD(mm)	56,09 ± 8,960
IVSdiameter(mm)	12,45 ± 7,115
PWdiameter(mm)	10,65 ± 1,768
LA diameter(mm)	48,30 ± 7,606
Diastolic pattern	
○ normal	126 (33,2%)
○ impaired relaxation	27 (7,1%)
○ intermediate	29 (7,6%)
○ restrictive	67 (17,6%)
○ monophasic	104 (27,4%)
MR grade	
○ 1 (mild)	42 (11,0%)
○ 2 (moderate)	153 (40,3%)
MS grade	
○ 1	14 (3,7%)
○ 2	10 (2,6%)
Prosthetic AV valve	41 (10,6%)
AS grade	
○ 1	20 (5,3%)
○ 2	15 (3,9%)
AR grade	
○ 1	67 (17,6%)
○ 2	53 (13,9%)
RV dilatation	40 (10,5%)
RVEDD basal (mm)	40,17 ± 5,60
RVEDD mid (mm)	36,50 ± 4,23
TAPSE (mm)	18,52 ± 3,93
TR grade	
Pericardial effusion	
○ mild	4 (1,0%)
○ moderate	7 (1,8%)
○ severe	0
**Laboratory exams**	
C-reactive protein (mg/L)	23,12 ± 39,33
WBC (10^9/L)	9,82 ± 8,43
Hematocrit (%)	37,01 ± 6,46
Albumin (g/L)	39,57 ± 8,11
Sodium (mmol/L)	139,70 ± 4,01
Potassium (mmol/L)	4,12 ± 0,59
Chloride (mmol/L)	102,04 ± 7,21
AST (UI/L)	78,05 ± 395,89
ALT (UI/L)	59,68 ± 215,41
Total bilirubin (mg/dL)	0,91 ± 1,99
INR	1,33 ± 0,61
Total cholesterol (mg/dl)	139,11 ± 40,63

Abbreviations: AR = aortic regurgitation; AS = aortic stenosis; AST = aspartate aminotransferase; AV = aortic valve; CKD = chronic kidney disease; COPD = chronic obstructive pulmonary disease; CRTP = cardiac resynchronization therapy pacemaker; CRTD = cardiac resynchronization therapy defibrillator; DBP = diastolic blood pressure; EDD = end-diastolic diameter; EDV = end-diastolic volume; ICD = implantable cardioverter-defibrillator; INR = international normalized ratio; IVC = inferior vena cava; IVS = interventricular septum; MAP = mean arterial pressure; MR = mitral regurgitation; MS = mitral stenosis; MV = mitral valve; NYHA = New York Heart Association; PM = pacemaker; PW = posterior wall; RAASis = renin–angiotensin–aldosterone system inhibitors; RV = right ventricle; RVEDD = right ventricular end-diastolic diameter; SD = standard deviation; SGLTis = sodium-glucose cotransporter inhibitors; TAVR = transcatheter aortic valve replacement; TR = tricuspid regurgitation; TV = tricuspid valve; WBC = white blood cells.

Supplementary Table 2. Univariate Analysis of Clinical and Echocardiographic Characteristics Stratified by the Presence of Chest X-Ray Congestion

Variable	absence of congestion at lung X ray N=106	presence of congestion at lung X ray N=274	p-value	Odds ratio (CI 95%)
**Demographics and comorbidities**				
Female sex	35 (33%)	97 (35%)	0,705	1,096 (0,681 -1,763)
Age at inclusion (years), mean±SD	75,1±11	76,7±11,3	0,22	1,012 (0,993-1,032)
Height (cm), mean±SD	168,7±8,5	167,2±9,4	0,278	0,984 (0,954-1,013)
Weight (kg), mean±SD	79,6±19,3	72,8±16,2	0,006	0,978 (0,963-0,994)
Smoker active/former	20 (18%)	71 (25%)	0,309	1,347 (0,758-2,394)
Hypertension	59 (55%)	200 (72%)	0,029	1,82 (1,065-3,113)
Dyslipidemia	50 (47%)	151 (55%)	0,61	1,136 (0,695-1,857)
Diabetes mellitus	34 (32%)	91 (33%)	0,672	0,898 (0,544-1,48)
Peripheral artery disease	19 (17%)	51 (18%)	0,774	0,917 (0,506-1,66)
Prior stroke/transitory ischemic attack	14 (13%)	39 (14%)	0,912	0,963 (0,495-1,874)
Prior AF	43 (40%)	109 (39%)	0,363	0,798 (0,49-1,298)
Permanent AF	23 (21%)	65 (23%)	0,682	1,12 (0,652-1,921)
Prior MI	21 (19%)	83 (30%)	0,118	1,558 (0,893-2,716)
Prior CAD diagnosis	29 (27%)	93 (33%)	0,491	1,198 (0,717-2)
Prior PCI	27 (25%)	72 (26%)	0,692	0,899 (0,53-1,525)
Prior CABG	3 (2%)	23 (8%)	0,097	2,833 (0,829-9,68)
Prior valve surgery	9 (8%)	28 (10%)	0,798	1,031 (0,815-1,304)
Prior percutaneous valve intervention	8 (7%)	14 (5%)	0,105	0,687 (0,436-1,082)
Prior myocarditis	1 (0%)	1 (0%)	0,454	0,345 (0,021-5,579)
Known cardiomiopathy	16 (15%)	24 (8%)	0,02	0,482 (0,26-0,893)
Prior device implantation	28 (26%)	61 (22%)	0,184	0,845 (0,659-1,084)
COPD	13 (12%)	30 (10%)	0,479	0,776 (0,385-1,565)
CKD	37 (34%)	125 (45%)	0,234	1,346 (0,825-2,197)
Dialysis	1 (0%)	5 (1%)	0,615	1,741 (0,201-15,111)
History of cancer	14 (13%)	56 (20%)	0,231	1,482 (0,778-2,823)
Depressive disorder	9 (8%)	16 (5%)	0,22	0,585 (0,249-1,377)
Dementia	14 (13%)	35 (12%)	0,605	0,837 (0,427-1,643)
De novo HF	45 (42%)	136 (49%)	0,58	1,146 (0,707-1,86)
**Objective examination**				
NYHA class III/IV	56 (52%)	233 (85%)	<,001	2,674 (1,914-3,737)
Declive oedema	20 (18%)	141 (51%)	<,001	6,375 (3,607-11,269)
Lung rales	32 (30%)	137 (50%)	0,001	2,292 (1,377-3,814)
**Vital parameters**				
SBP (mmHg)	133,3±25,8	140,6±34,5	0,078	1,007 (0,999-1,015)
DBP (mmHg)	76,2±16,7	78,7±21	0,33	1,006 (0,994-1,019)
HR (bpm)	85,1±24,7	89,8±26,1	0,163	1,007 (0,997-1,017)
**Rhythm at ECG**				
AF/AT	38 (35%)	89 (32%)	0,181	0,712 (0,432-1,171)
**Echocardiogram**				
LVEF(%), mean±SD	41,3±13,1	36,9±13,3	0,005	0,976 (0,959-0,992)
EDV (mL), mean±SD	144,6±78,2	144,2±60,1	0,964	1 (0,996-1,004)
EDD(mm), mean±SD	55,7±8,7	56,2±9	0,713	1,005 (0,977-1,035)

IVS diameter (mm), mean±SD	12,1±2,8	12,5±8,1	0,641	1,014 (0,957-1,074)
PW diameter(mm), mean±SD	10,4±1,6	10,7±1,7	0,237	1,102 (0,938-1,295)
LA volume(mL), mean±SD	98,5±38,5	111,6±71,4	0,266	1,005 (0,996-1,014)
LA diameter (mm), mean±SD	49,4±6,6	47,8±7,9	0,361	0,973 (0,918-1,032)
Diastolic pattern > 2	36 (33%)	125 (45%)	0,68	1,029 (0,897-1,181)
Severe MR	29 (27%)	131 (47%)	<,001	2,528 (1,546-4,134)
Severe MS	1 (0%)	3 (1%)	0,922	1,120 (0,115-10,917)
Severe AS	8 (7%)	26 (9%)	0,505	1,325 (0,579-3,031)
Severe AR	0 (0%)	17 (6%)	0,998	
RVEDD basal (mm), mean±SD	39,3±5,5	40,5±5,6	0,495	1,038 (0,933-1,154)
RV dilatation	12 (11%)	28 (10%)	0,769	0,898 (0,438-1,841)
TAPSE (mm)	18,9±4,2	18,3±3,8	0,463	0,962 (0,866-1,067)
RV disfunzione	10 (9%)	37 (13%)	0,414	1,34 (0,664-2,703)
sPAP (mmHg)	40,6±12,3	55,8±10,4	<,001	1,13 (1,098-1,163)
CVP (mmHg)	7,1±3,6	12,2±3,9	<,001	1,406 (1,304-1,516)
Pericardial effusion (any grade)	4 (3%)	7 (2%)	0,993	1,003 (0,458-2,198)
**Laboratory exams**				
Ferritin (ng/mL)	158,2±171,9	232,5±344,1	0,161	1,002 (0,999-1,004)
Transferrin saturation (%)	18,6±10,9	19,3±13,1	0,758	1,004 (0,976-1,033)
Troponin T >14 ng/L	68	225	0,004	4,136 (1,570-10,894)
NTproBNP>3000 pg/mL	25 (23%)	164	<,001	5,655(3,212-9,958)
Creatinine(mg/dL)	1,5±0,9	1,7±1,1	0,226	1,168 (0,908-1,501)
C-reactive protein (mg/L)	66,2±42,2	74,7±45,9	0,226	1,005 (0,997-1,013)
WBC (10^9/L)	18,4±39,1	24,6±39,3	0,708	1,006 (0,973-1,041)
Hemoglobin(g/dL)	9,5±10,2	9,9±7,7	0,56	1,034 (0,925-1,156)
Hematocrit(%)	127,3±539,3	173,6±691,6	0,305	1,02 (0,982-1,06)
Albumin (g/L)	12±2,3	12,1±2,1	0,515	0,99 (0,959-1,021)
Sodium (mmol/L)	36,3±6,8	37,2±6,3	0,791	0,992 (0,932-1,055)
Potassium (mmol/L)	211,2±81,8	2502,3±20915,8	0,836	0,957 (0,632-1,449)
Chloride (mmol/L)	40,1±6,3	39,4±8,5	0,502	0,985 (0,943-1,029)
AST (UI/L)	33,78±44,582	92,44±454,317	0,174	1,005 (0,998-1,011)
ALT (UI/L)	139,8±3,6	139,6±4,1	0,02	1,013 (1,002-1,024)
Total bilirubin (mg/dL)	4,1±0,5	4,1±0,6	0,334	0,94 (0,829-1,066)
INR	102,5±4,6	101,8±7,8	0,937	0,983 (0,651-1,486)

Abbreviations: AF = atrial fibrillation; ALT = alanine aminotransferase; AR = aortic regurgitation; AS = aortic stenosis; AST = aspartate aminotransferase; AV = aortic valve; CAD = coronary artery disease; CKD = chronic kidney disease; COPD = chronic obstructive pulmonary disease; CRTP = cardiac resynchronization therapy pacemaker; CRTD = cardiac resynchronization therapy defibrillator; CVP = central venous pressure; DBP = diastolic blood pressure; EDD = end-diastolic diameter; EDV = end-diastolic volume; EF = ejection fraction; HR = heart rate; ICD = implantable cardioverter-defibrillator; INR = international normalized ratio; IVC = inferior vena cava; IVS = interventricular septum; LA = left atrium; LVEF = left ventricular ejection fraction; MAP = mean arterial pressure; MR = mitral regurgitation; MS = mitral stenosis; MV = mitral valve; NTproBNP = N-terminal pro-B-type natriuretic peptide; NYHA = New York Heart Association; PM = pacemaker; PW = posterior wall; RAASis = renin–angiotensin–aldosterone system inhibitors; RV = right ventricle; RVEDD = right ventricular end-diastolic diameter; SBP = systolic blood pressure; SD = standard deviation; SGLTis = sodium-glucose cotransporter inhibitors; sPAP = systolic pulmonary artery pressure; TAVR = transcatheter aortic valve replacement; TR = tricuspid regurgitation; TnT = troponin T; TV = tricuspid valve; WBC = white blood cells.
